# Prognostic value of programmed death-ligand 1 in sarcoma: a meta-analysis

**DOI:** 10.18632/oncotarget.19168

**Published:** 2017-07-11

**Authors:** Zhenhua Zhu, Zheng Jin, Mei Zhang, Yajun Tang, Guang Yang, Xiaowei Yuan, Jihang Yao, Dahui Sun

**Affiliations:** ^1^ Department of Orthopaedic Trauma, The First Hospital of Jilin University, Changchun, China; ^2^ Department of Immunology, College of Basic Medical sciences, Jilin University, Changchun, China; ^3^ College of Chemistry, Jilin University, Changchun, China

**Keywords:** programmed death-ligand 1, sarcoma, prognosis, survival, meta-analysis

## Abstract

**Background:**

The prognostic role of programmed death-ligand 1 (PD-L1) in sarcoma remains controversial. We performed a meta-analysis so as to investigate the impact of PD-L1 on clinicopathlogical findings and survival outcomes in sarcoma.

**Materials and Methods:**

A comprehensive search in PubMed, Embase and the Cochrane Library was conducted for relevant studies. The odds ratios or hazard ratios, at 95% confidence intervals were used as measures for investigation of the correlation between PD-L1 expression and clinicopathlogical features or survival outcomes.

**Results:**

Fourteen eligible studies comprising 868 patients were selected for analysis. Pooled hazard ratios indicated that the association of PD-L1 expression with overall survival in bone sarcoma (osteosarcoma and chondrosarcoma) patients was statistically significant (1.987, 95% CI: 1.224–3.224, *p* = 0.005), as was its association with event-free survival in bone and soft-tissue sarcoma patients (3.868, 95% CI: 2.298–6.511, *p* = 0.000). Additionally, the expression of PD-L1 was positively correlated with the infiltration of programmed death 1 (PD-1) positive T-lymphocytes (OR: 4.012, 95% CI: 2.391–6.733, *p* = 0.000).

**Conclusions:**

Our meta-analysis indicated that high PD-L1 expression is likely to be a negative factor for patients with sarcomas and that it predicts worse survival outcomes.

## INTRODUCTION

Sarcomas are malignant tumors that are characterized by a wide diversity of subtypes with various cytogenetic profiles. Despite major treatment breakthroughs, standard treatment modalities combining chemotherapy, radiotherapy and surgery have failed to improve overall survival [1]. Together, sarcomas affect approximately 11,000 individuals in the United States each year and approximately 200,000 worldwide, arising from multiple lineages and ranging from indolent tumors to those which are highly invasive and metastatic [2]. Recent data reports 5-year survival rates of 66% for bone and soft tissue sarcomas, 53.9% for osteosarcoma, 75.2% for chondrosarcoma and 50.6% for Ewing's sarcoma [3].

Immune escape is regarded as an important biological process for primary cancer growth and metastasis. Programmed death 1 (PD-1) and programmed death-ligand 1 (PD-L1) have proved to play a predominant role in cancer immune surveillance [4, 5]. In solid tumors, PD-1/PD-L1 blockade has achieved profound progress, showing vast potential for tumor therapy [6–9]. In order to improve survival outcome of patients with sarcoma, the association between PD-L1 expression and prognosis of sarcoma patients has been examined, but the results have been controversial.

In the present study, a meta-analysis was conducted to evaluate the association between PD-L1 expression and clinicopathological characteristics or survival outcomes in bone and soft-tissue sarcoma patients.

## RESULTS

### Search results

**Figure 1:**
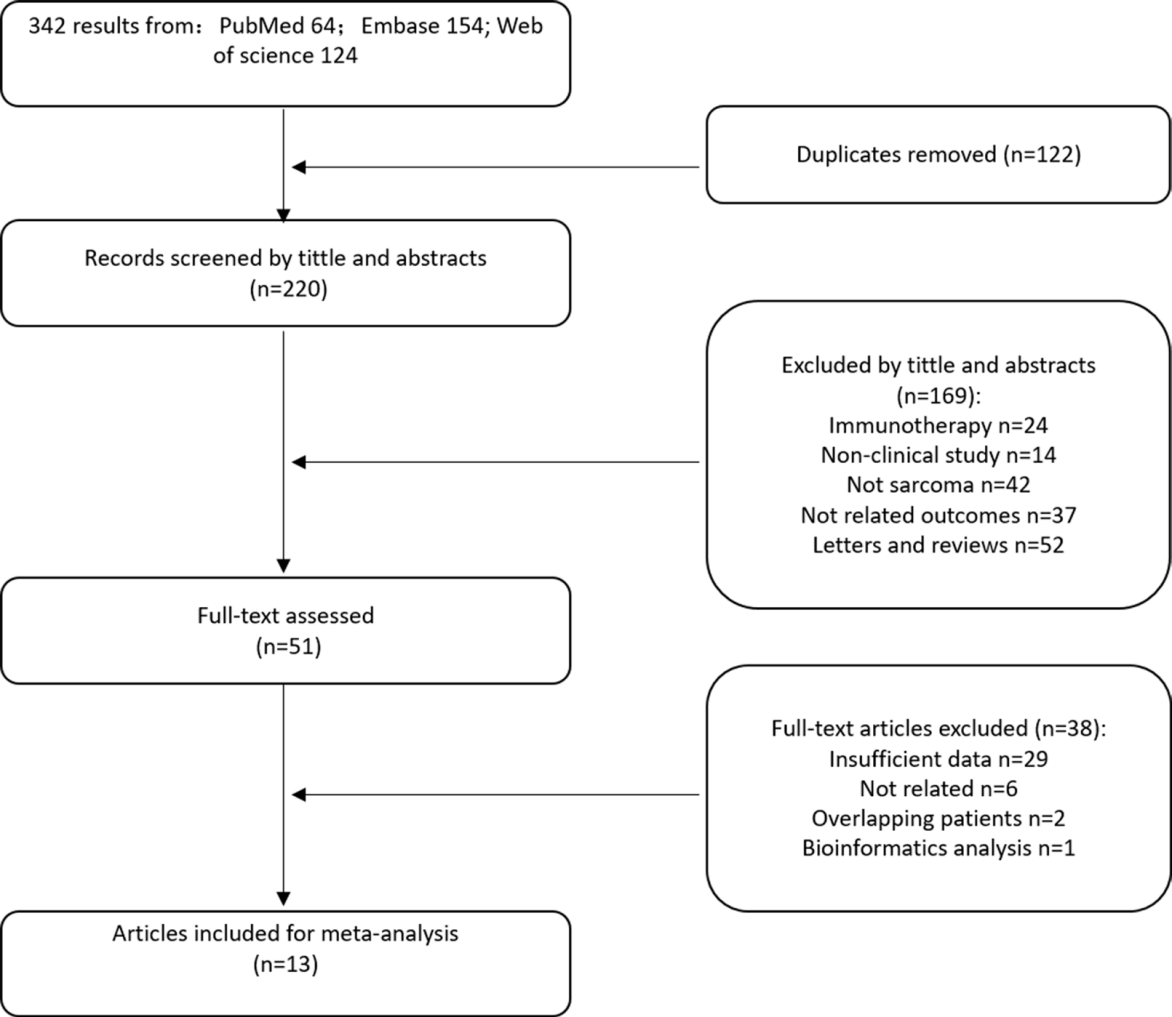
Flow chart for selection of studies

For primary retrieval, a total of 342 citations were identified by searching through three electronic databases, including 64 citations in PubMed, 154 citations in Embase and 124 citations in Web of Science. After removing 122 duplicates, the remaining 220 records were screened during the initial filtering, in which 169 records were excluded after viewing the titles and abstracts. From the remaining 51 articles a further 38 were excluded after viewing the full-text, including 29 articles with insufficient data, two with overlapping patients, six articles that were not relevant and one bioinformatics analysis. Eventually, 13 manuscripts [10–22] containing 14 independent studies were enrolled in the meta-analysis (Figure 1).

### Study characteristics

All studies that were included were published between 2013 and 2017, the number of patients in each study ranging from 13 to 137. The proportion of patients expressing PD-L1 ranged from 6% to 75%. The basic characteristics of the 14 eligible studies are summarized in Table 1. Among them, 12 studies assessed the prognostic significance of PD-L1 expression and 13 focused on investigating its clinicopathological significance. Osteosarcoma was the most researched in seven studies. The histological subtypes discussed in the other studies were a mixed type of soft-tissue sarcoma in three studies, a mixed type of bone and soft-tissue sarcoma, chondrosarcoma, and angiosarcoma in three independent studies.

**Table 1:** Features of included studies

No.	Study	Patient source	Study period	Follow-up(month)	Histological type	Median age	Number of pateints^a^	PD-L1+ patients (%)	Effect size	Patients included^b^	NOS score
1	Chowdhury [10]	UK	—	33 (3–200)	Ewing sarcoma Rahbdomyosarcoma Osteosarcoma	8 (0.8–16.6)	59	59.3%	NA	0	7
2	Costa [11]	Brazil	1997–2014	15 (2–156)	Oral osteosarcoma	28 (23–65)	13	69.2%	OS (available data)	13	6
3	D’Angelo [12]	USA	2004–2013	10.24	STS	46 (22–76)	47	8.5%	OS (p value)	47	5
4	Honda [13]	Japan	1996–2016	20 (3–100)	Cutaneous angiosarcoma	74.5	106	30.2%	OS (survival curve)	92	7
5	Kim, C [14]	South Korea	1994–2013	33.8 (3.8–84.8)	STS	26 (1–78)	82	42.7%	OS (provided in the paper );	82	7
									RFS (survival curve)	82	
6	Kim, J [15]	South Korea	1998–2011	35 (1–175)	STS	—	105	64.8%	OS (provided in the paper)	105	8
									EFS (provided in the paper)	105	
7	Koirala corhort1 [16]	USA	—	84 (4–150)	Osteosarcoma	18	51	5.9%	EFS (survival curve)	51	7
8	Koirala corhort2 [16]	USA	—	54 (15–100)	Osteosarcoma	16	41	29.3	EFS (survival curve)	41	7
9	Kostine [17]	Europe	—	17 (1–60)	Chondrosarcoma	—	137	14.6%	OS (survival curve)	20	6
10	Lussier [18]	USA	—	—	Osteosarcoma	15 (9–21)	16	75.0%	NA	0	4
11	Palmerini [19]	Italy	2001–2006	96(12–156)	Osteosarcoma	16 (4–39)	86	14.0%	OS (p value)	86	8
12	Paydas [20]	Adana	—	30 (4–310)	Sarcoma	45 (17–85)	66	30.3%	OS (available data)	55	6
									PFS (available data)	41	
13	Shen, J [21]	USA	—	36 (1–200)	Osteosarcoma	29 (6–75)	37	27.0%	OS (available data)	37	6
14	Sundara [22]	Netherlands	1998–2011	56 (14–117)	Osteosarcoma	18 (7–70)	22	18.2%	OS (survival curve)	22	8
									DFS (survival curve)	22	

^a^:total number of patients included in each study; ^b^: number of patients included in survival analysis.

NOS: Newcastle-Ottawa Scale; STS: soft-tissue sarcoma; NA: not available; OS: overall survival; RFS: recurrence-free survival; EFS: event-free survival; DFS: disease-free survival; PFS: progress-free survival.

To detect the expression of PD-L1, all studies used immunohistochemistry, except for one study [21], which used quantitative real-time PCR, but the proportion of PD-L1 expression in that study was consistent with the others. The detailed methodologies used to detect PD-L1 are summarized in Table 2. When it came to survival outcomes, different effect sizes and outcomes were reported, including overall survival (OS) reported in 10 studies, event-free survival (EFS) reported in three, and recurrence-free survival (RFS), disease-free survival (DFS) and progress-free survival (PFS) reported independently in three studies. Additionally, in Koirala's study [16], two cohorts of patients were reported, PD-L1 was assessed with distinct operations and the survival curves were reported independently, so they have been statistically analyzed as two individual studies. All studies were followed for more than five years.

**Table 2:** Methods for PD-L1 detection

Study	Method	Antibody type	Antibody dilution	Antibody source	Cutoff value
Chowdhury	IHC	——		Abcam	> 5% of tumor cells
Costa	IHC	Monoclonal	1: 400	Cell signaling	Total score > 2^a^
D’Angelo	IHC	——	——	DAKO	> 1% of tumor cells
Honda	IHC	Monoclonal	——	Spring bioscience	> 5% of tumor region
Kim, C	IHC	Monoclonal	1: 100	R&D system	Total score > 2^a^
Kim, J	IHC	——	1: 100	Santa Cruz	Total score > 8^b^
Koirala	IHC	Monoclonal	1: 50	——	> 1% of tumor cells
kostine	IHC	Monoclonal	1: 400	Cell signaling	> 1% of cells
Lussier	IHC	Monoclonal	1:200	Abcam	> 1 cell/high-power field
Palmerini	IHC	——	——	——	——
Paydas	IHC	——	——	AM26531AF-N Acris	> 5% of cells
Shen, J	qRT-PCR				
Sundara	IHC	Monoclonal	1: 400	Cell signaling	≥ 1% of cells

IHC: immunohistochemistry

qRT-PCR: quantitative real time polymerase chain reaction

^a^ :Total score was calculated by adding a score of staining percentage to another score of staining intensity. The area of staining was scored as 0 (no tumor cells stained), 1 (< 25% of cells stained), 2 (≥ 25% of cells stained). Staining intensity was graded as 0 (no staining), 1 (weak staining), 2 (moderate staining), 3 (strong staining)

^b^:Total score was calculated by summing up the proportion score and intensity score of two different tissue microarray (TMA) cores. The area of staining was scored as 0 (0–10% of the cells stained), 1 (11–33% of the cells stained), 2 (34–66% of the cells stained), and 3 (67–100% of the cells stained). The staining intensity scored as 0 (no staining), 1 (weak staining), 2 (intermediate staining), and 3 (strong staining).

### Correlation between PD-L1 expression and overall survival

**Figure 2:**
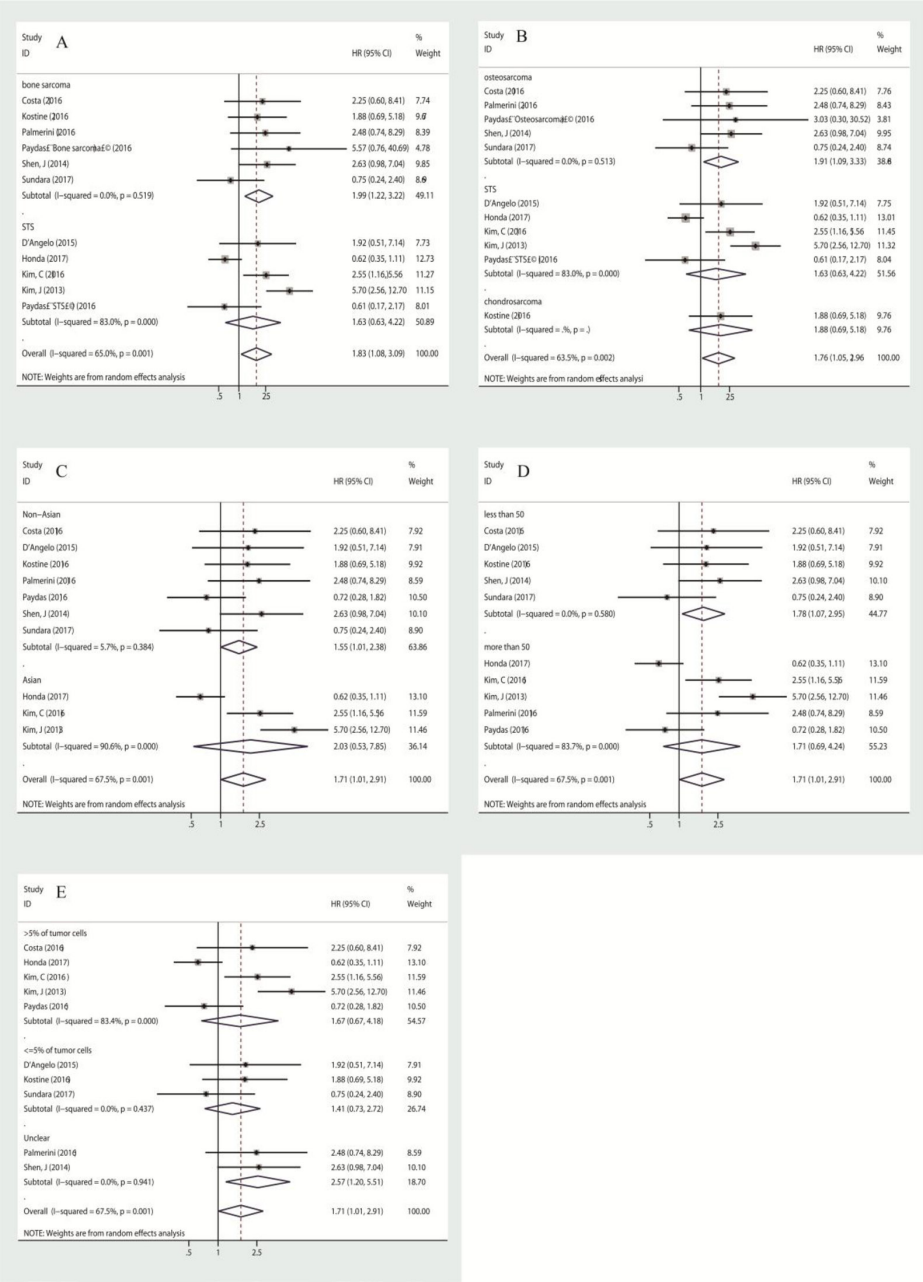
**Subgroup analysis of association between PD-L1 expression and overall survival**, stratified by histological subtype (**A**, **B**), by patient source (**C**), by the number of patients included in the study (**D**), by cut-off (**E**).

A total of 10 studies with 559 patients were enrolled in the analysis of overall survival. As a significant degree of heterogeneity was detected (I^2^ = 67.5%, *p* = 0.001), subgroup analysis was performed to clarify the source. In subgroup analysis stratified by histological subtype, the pooled hazard ratio (HR) estimate for overall survival was 1.987 (95% CI: 1.224–3.224, *p* = 0.005) in patients with bone sarcoma (osteosarcoma and chondrosarcoma) with no heterogeneity (I^2^ = 0.0%, *p* = 0.519), and 1.625 (95% CI: 0.627–4.216, *p* = 0.318) in patients with soft-tissue sarcomas with high heterogeneity (I^2^ = 83.0%, *p* = 0.000) (Figure 2A). As for individual histological type, PD-L1 was a poor prognostic factor for osteosarcoma with a pooled HR of 1.908 (95% CI: 1.093–3.331, *p* = 0.023) (Figure 2B). In the subgroup analysis stratified by country, pooled HR was 1.546 (95% CI: 1.005–2.379, *p* = 0.047) for non-Asian patients, with low heterogeneity (I^2^ = 5.7%, *p* = 0.384), and 2.033 (95% CI: 0.526–7.849, *p* = 0.303) for Asian patients with high heterogeneity (I^2^ = 90.6%, *p* = 0.000) (Figure 2C). When stratified by the number of patients, pooled HR was 1.779 (95% CI: 1.072–2.951, *p* = 0.026) for studies of which the patients included were less than 50, with no heterogeneity (I^2^ = 0.0%, *p* = 0.580) (Figure 2D). To clarify the impact of a different cut-off of PD-L1 expression on the results, we conducted subgroup analysis stratified by cut-off. Both when cut-off <= 5% and in studies without clear cut-off the heterogeneity was negligible, but there was no statistical significance between PD-L1 expression and overall survival (Figure 2E). As for the two studies [19, 21] without any definition of cut-off, exclusion of either did not change the conclusion for bone sarcoma (Supplementary Figure 1A, 1B), but the conclusion for non-Asian patients did change (Supplemental data, Figure 1C, 1D).

### Correlation between PD-L1 expression and event-free survival

**Figure 3:**
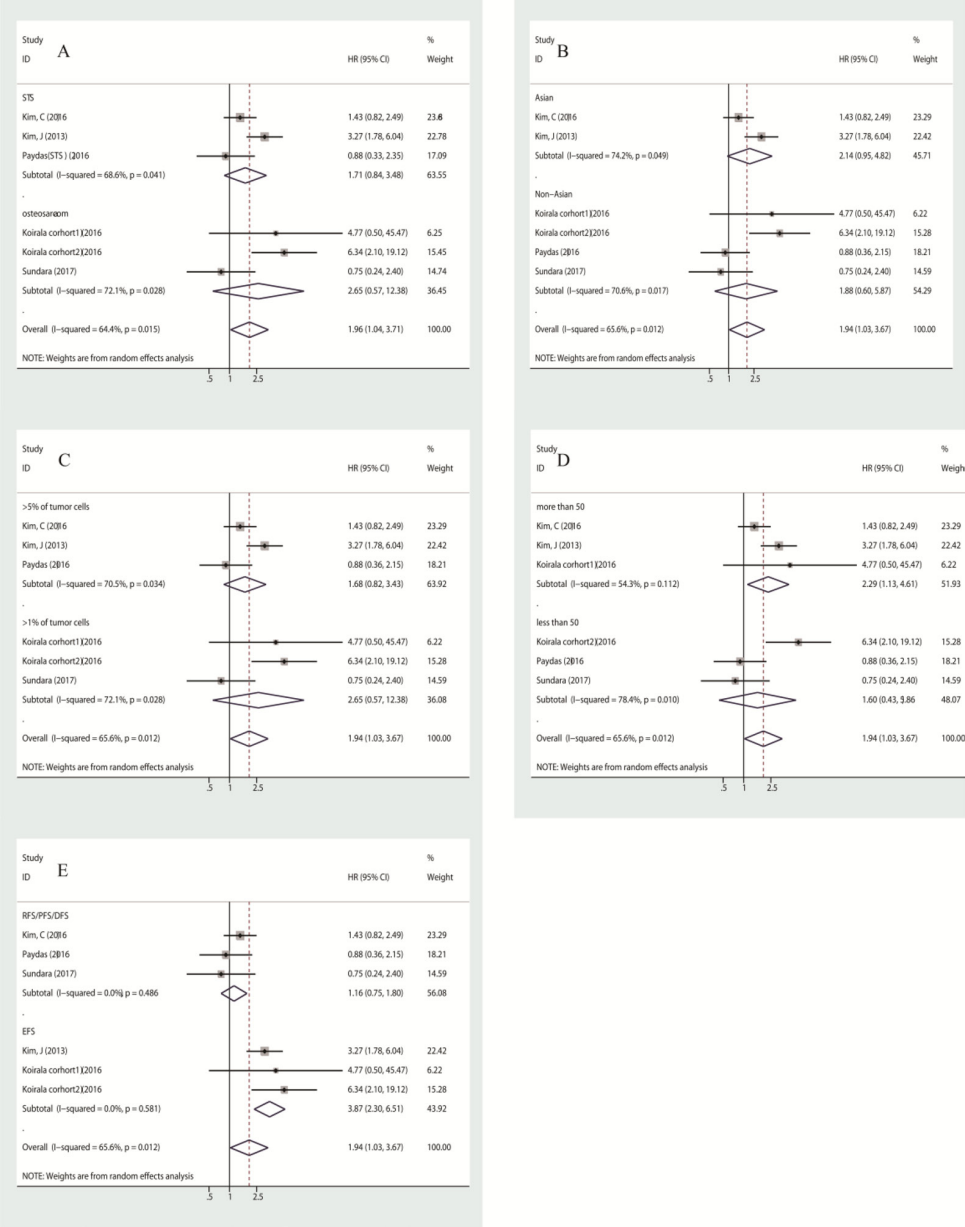
**Subgroup analysis of association between PD-L1 expression and event-free survival**, stratified by histological subtype (**A**), by patient source (**B**), by cut-off (**C**), by number of patients (**D**), by effect size (**E**).

Three studies assessed the correlation between PD-L1 and event-free survival. An event was defined as local recurrence, later distant metastasis or death, which is in accordance with the meaning of DFS, RFS or PFS. We combined the HRs of EFS, DFS, RFS and PFS, this pooled HR taken as the effect size to assess survival outcome. Due to medium heterogeneity (I^2^ = 65.6%, *p* = 0.012) in the group, a random-effects model was used to assess it, with a pooled HR of 1.943 (95% CI: 1.028–3.674, *p* = 0.041). In subgroup analyses, whether stratified by histological subtype, country or cut-off, heterogeneity was not excluded (Figure 3A–3C). When stratified by numbers of patients, pooled HR was 2.286 (95% CI: 1.133–4.612, *p* = 0.021) for group of which patients were more than 50, with medium heterogeneity (I^2^ = 54.3%, *p* = 0.112) (Figure 3D). However, when they were stratified by effect size, both subgroups, including the EFS group and other effect groups including PFS, DFS and RFS, showed negligible heterogeneity. The HR of pooled EFS was 3.868 (95% CI: 2.298–6.511, *p* = 0.000) with no heterogeneity (I^2^ = 0.0%, *p* = 0.581), and the HR of pooled RFS/DFS/PFS was 1.164 (95% CI: 0.753–1.800, *p* = 0.495) with no heterogeneity (I^2^ = 0.0%, *p* = 0.486) (Figure 3E). Overall, PD-L1 was poorly prognostic for event-free survival in both bone and soft-tissue sarcoma patients.

### Correlation between PD-L1 expression and tumor clinicopathlogical features

**Figure 4:**
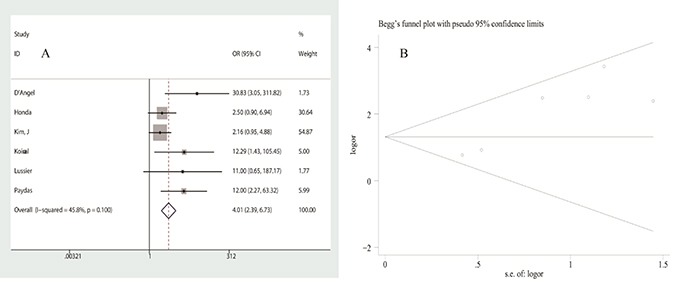
Analysis of the association between PD-L1 expression and PD-1+ T-lymphocyte infiltration (**A**) forest plot of ORs, (**B**) publication bias.

Five studies assessed the correlation of PD-L1 expression with infiltration of PD-1 positive T-lymphocytes with low heterogeneity (I^2^ = 45.8%, *p* = 0.100). Using a fixed-effects model, pooled OR was calculated to be 4.012 (95% CI: 2.391–6.733, *p* = 0.000), indicating that PD-L1 expression was significantly associated with the infiltration of PD-1 positive lymphocytes (Figure 4A). Nevertheless, it should be noted that a significant publication bias was found in the analysis (Figure 4B). Furthermore, we failed to find a significant correlation between age, gender, metastasis, tumor site or size or grade, and the infiltration of CD3+/CD4+/CD8+ T-lymphocytes. Data is shown in Table 3.

**Table 3:** Association between PD-L1 expression and clinical features

Association between PD-L1 expression and clinical features	NO. of study	OR 95% CI	z, P (OR)	Heterogeneity test (I^2^, *P* bias)	Publication Bias (Egger test)(t, *P*)	Pooling model
Age < 20 VS Age ≥ 20	12,13	2.228 (0.612–8.110)	1.22, 0.224	0.0%, 0.480		fixed
Male VS Female	1,2,4,5,6,12,13	1.250 (0.849–1.842)	1.13, 0.258	35.0%, 0.161	−0.68, 0.525	fixed
Initial distant metastasis at diagnosis YES VS NO	2,5,6	1.681 (0.846–3.340)	1.48, 0.138	31.8%, 0.231	−0.60, 0.655	fixed
tumor site limbs VS others	4,5,12	1.537 (0.474–4.987)	0.72, 0.474	57.4%, 0.095	−0.30, 0.813	random
Size >5 cm VS ≤ 5 cm	2,6,12	1.032 (0.513–2.076)	0.09, 0.929	0.0%, 0.653	−1.03, 0.490	fixed
Grade 2, 3 VS 1	5,6	2.670 (0.884–8.064)	1.74, 0.082	54.8%, 0.137		random
CD4+ T lymphocytes infiltration YES VS NO	3,7	2.452 (0.689–8.731)	1.38, 0.166	0.0%, 0.942		fixed
CD8+ T lymphocytes infiltration YES VS NO	1,3,7	2.269 (0.615–8.364)	1.23, 0.218	59.3%, 0.086	6.24, 0.101	random
PD-1+ T lymphocytes infiltration YES VS NO	3,4,6,7,10,12	4.012 (2.391–6.733)	5.26, 0.000*	45.8%, 0.100	4.76, 0.009*	fixed

OR: odds ratio.

*:statistical significance.

### Sensitivity analysis and evaluation of Publication bias

**Figure 5:**
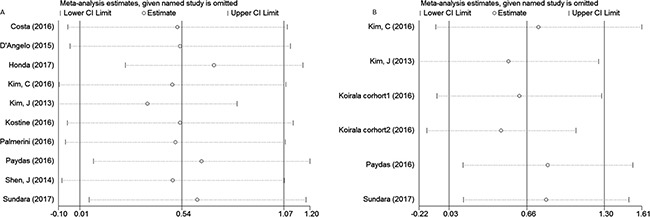
Sensitivity analysis for the association between PD-L1 expression and overall survival (**A**), event-free survival (**B**).

**Figure 6:**
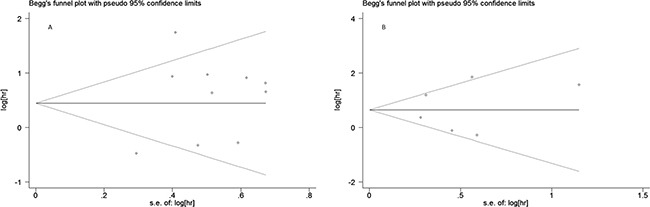
Begg's funnel plot for publication bias analysis (**A**) association between PD-L1 expression and overall survival, (**B**) association between PD-L1 expression and event-free survival.

Sensitivity analysis was conducted to evaluate the influence of a unique study on pooled HR. As shown in Figure 5A and 5B, the pooled HRs float within the 95% confidence interval, indicating that the results are stable and reliable. To assess publication bias, a Begg's funnel plot was used, in which log HRs were plotted against their corresponding standard errors (SEs). No apparent asymmetry was observed in the funnel plot through visual evaluation (Figure 6A, 6B). Furthermore, Egger's test which provides statistical estimation of publication bias found no evidence of it (*p* = 0.350 in OS, *p* = 0.848 in EFS), indicating that such bias was not present within the studies.

## DISCUSSION

Sarcoma is a rare heterogeneous disease with more than 70 different subtypes, accounting for 1% of all cancers diagnosed in the United States each year [12]. Metastasis is common among these patients, and cures using traditional therapies for sarcomas have been stagnant for decades. In contrast, there have been tremendous breakthroughs in other malignancies by manipulating the immune system with checkpoint inhibitors [12]. Therefore, there are high expectations for immunotherapy when the field matures and a better understanding of its mechanism of action has been gained [1].

The interaction between PD-L1 in tumor cells and PD -1 in T-lymphocytes negatively regulates the tumor-killing function of T-lymphocytes and protects tumor cells from the host immune system. Recently, much attention has been paid to PD-L1 expression in various solid tumors, due to the FDA's approval for anti-PD-1 antibodies in non-small cell lung cancer and melanoma with good efficacy and safety. Several clinical trials have reported that therapies targeting PD-1 and its ligand (PD-L1) improve patient outcomes, while tumor response has been related to PD-L1 expression [23, 24]. Recent studies have indicated that high expression of PD-L1 is associated with poor prognosis in non-small cell lung cancer, kidney cancer, bladder cancer, prostate and gastric cancer [25–29]. However, for patients with osteosarcoma, the association between the expression of PD-L1 and their survival outcomes remains controversial. Multiple studies have indicated that PD-L1 expression is associated with a significant poor survival outcome [10, 11, 14–16], while one study reported the opposite effect [13] and other studies have shown no association [12, 17, 19–22].

In the current meta-analysis, we combined 14 studies related to prognosis and clinicopathology of PD-L1 expression in sarcoma patients. For all sarcoma patients, we found that the expression of PD-L1 was significantly associated with poor event-free survival. In contrast, PD-L1 expression was significantly associated with overall survival in bone sarcoma (osteosarcoma and chondrosarcoma) rather than soft tissue sarcoma. As for patient ethnicity, there was a significant association between PD-L1 expression and poor overall survival in non-Asian patients, but one should draw conclusions carefully, in consideration of bias caused by the cut-off. Moreover, PD-L1 expression predicts poor event-free survival in bone and soft tissue sarcoma.

We found that PD-L1 expression was significantly associated with the infiltration of PD-1 positive T-lymphocytes, indicating that an adaptive immune resistance mechanism may be occurring [30]. In this case, PD-L1 was likely upregulated because of a negative feedback loop that follows the activation of cytotoxic cells [31], a correlation that was confirmed in mRNA expression levels in soft-tissue sarcoma patients by Bertucci [31]. Therefore, the positive correlation between PD-L1 expression and PD-1 positive lymphocytes assessed here suggests that immune checkpoint PD-L1/PD-1 blockade holds great potential for improving the survival of sarcoma patients. In this case, blockade of PD-L1 might help reactivate inhibited T cells to increase the antitumor immune response.

Significant heterogeneity was noted in some analyses in the current study. This could have arisen from various sources. Firstly, the methods of PD-L1 measurement varied among these studies. Although the most common method was IHC, they did not use the same antibody and the dilution was also different. Because the type and the concentration of antibody can affect the sensitivity of IHC, differences also exist in the definition of PD-L1 expression. Secondly, the region where PD-L1 was expressed was not clear. Limited studies distinguished whether the PD-L1-positive cell region was from the tumor cells or the tumor microenvironment [12, 19, 20], whereas others have taken them together as tumor tissue PD-L1 expression [10, 11, 13–18, 21, 22]. It appears from the studies that the nature of PD-L1 expression differed within the same cohort of patients. In order to reduce the error among studies, we assessed as positive expression of PD-L1 in both the tumor and tumor environment. Furthermore, the association between overall survival and PD-L1 expression whether in the tumor or its microenvironment was found to have no statistically significant difference, due to the limited number of studies (Supplementary Figure 2).

Another potential source of bias may be linked to the method of calculation of the HRs. When the data from multivariate or univariate survival analyses were reported, we used them directly. If the HRs were not provided explicitly, we calculated them from outcome data available in the articles. If this was impracticable, we extrapolated them from the Kaplan-Meier curves or quoted *p* values using Tierney's methods [32], which was less reliable than using the HRs given directly in the papers. To minimize statistical bias brought about by comparing data from univariate and multivariate analyses, we preferentially used HRs from univariate analyses, except for one study [13], of which only the results of multivariate analysis were provided. Our conclusions of STS analysis did not change whether this study was excluded or not (Supplementary Figure 3).

Publication bias is another concern in all forms of meta-analysis because only positive results tend to be published in journals. To minimize publication bias, we attempted to conduct literature searches as completely as possible, using Web of Science, PubMed, Embase and the Cochrane Library. No significant publication bias was found in this meta-analysis, except for the analysis of tumor infiltration of PD-1 positive lymphocytes.

Additionally, the definition of bone sarcoma and soft-tissue sarcoma may bring heterogeneity to the whole meta-analysis. In other papers, osteosarcoma, Ewing sarcoma and chondrosarcoma have been included in the category of bone sarcoma [33]. However, in the papers included in this meta-analysis, two studies [14, 15] included Ewing sarcoma together with other soft tissue sarcomas and defined them all as STS, with 24 being the total number for such Ewing sarcoma patients. As survival outcomes were provided as a whole, we could not extract them from the pooled results. To minimize statistical bias, we analyzed whether exclusion of either or both of these studies would alter our conclusion of STS. However, we found that it did not (Supplementary Figure 4).

Therefore, the results of the current meta-analysis should be interpreted with caution and should be confirmed in well-designed prospective studies with appropriate multivariate analyses.

In conclusion, this meta-analysis demonstrated that PD-L1 expression may be an effective predictive factor of poor prognosis and clinicopathological features for bone and soft tissue sarcomas. Furthermore, PD-L1 expression could be used to identify a subgroup of patients who would potentially benefit from targeted therapy against PD-1 or PD-L1.

## MATERIALS AND METHODS

### Data source

A systematic literature search of PubMed, Embase and the Cochrane Library was undertaken with no language restrictions (last search, April 2017). The strategy used was to search for the following words in relevant literature: (“sarcoma” OR “soft tissue sarcoma” OR “bone sarcoma” OR “osteosarcoma” OR “chondrosarcoma” OR “Ewing sarcoma” OR “leiomyosarcoma” OR “angiosarcoma” OR “synovial sarcoma” OR “malignant fibrous histiocytoma” OR “liposarcoma” OR “rhabdomyosarcoma”) AND (“CD274” OR “B7-H1” OR “PD-L1” OR “programmed cell death 1 ligand 1 protein”).

### Inclusion criteria

Patients and studies had to fulfill the following criteria to be included in the analysis: (1) Patients with pathologically confirmed sarcoma who underwent detection of PD-L1 in their tumor tissue; (2) Studies evaluating the relationship between PD-L1 and clinical features and/or survival outcomes. Studies were excluded if they: (1) were case reports, reviews or letters; (2) comprised overlapping sarcoma patients; (3) had insufficient information such that the correlation of clinical features or survival outcomes could not be extracted. When there were multiple publications regarding the same group of patients, only the most recent publication was included. Two researchers (Zhenhua Zhu, Zheng Jin) screened the titles and abstracts of all the searched articles and verified that the studies met the inclusion criteria for subsequent analysis.

### Data extraction

The final articles that were included were assessed independently by two researchers (Zhenhua Zhu, Zheng Jin). Data extracted included: (1) basic information including first author, year of publication, study period, follow-up duration; (2) information about the patients and tumors, including patient source, number of patients, gender, age, number of patients with PD-L1 expression, histological type of tumor, tumor site, tumor size, grade at diagnosis, tumor-infiltrating lymphocytes; (3) outcome measures including metastasis, recurrence, survival data, Kaplan-Meier curves, *p* values and events; and (4) other variables including the methods of quantitative PD-L1 measurement, the definition of positivity (the cut-off value) and the antibody's type, source and dilution used for immunohistochemistry (IHC). Disagreements were settled by consultation.

### Quality assessment

In accordance with the Newcastle-Ottawa Scale (http://www.ohri.ca/programs/clinical_epidemiology/nos_manual.pdf), assessment of quality mainly focused on selection (representativeness, selection of the non-exposed, ascertainment of exposure and outcome of interest), comparability, and the outcome (assessment and follow-up) of the original studies. Assessment was carried out independently by two researchers (Zhenhua Zhu, Zheng Jin).

### Statistical analysis

For the quantitative aggregation of the survival results, hazard ratios (HRs) and their 95% confidence intervals (CIs) were combined to give the effect size. Additionally, the pooled ORs and their corresponding 95% CIs were utilized to quantitatively determine the association between PD-L1 and clinicopathological characteristics of patients. Statistical heterogeneity among studies was assessed using Cochran's *Q* test and Higgins I^2^ statistic. A fixed-effects model (Mantel-Haenszel method) was used to calculate parameters when no obvious heterogeneity existed among studies (I^2^ > 50% suggested high heterogeneity). Otherwise, a random-effects model was utilized. Publication bias was measured using funnel plots and Egger's test. All statistical analyses were conducted using STATA version 12.0 (STATA corp., College Station, TX.)

## SUPPLEMENTARY FIGURES



## Abbreviations

PD-L1: programmed death-ligand 1
PD-1: programmed death 1
HR: hazard ratio
OR: odds ratio
STS: soft tissue sarcoma
OS: overall survival
EFS: event-free survival
PFS: progress-free survival
RFS: recurrence-free survival
DFS: disease-free survival
IHC: immunohistochemistry
qRT-PCR: quantitative real time polymerase chain reaction
