# Supplementary material for: Prognostic value of programmed death-ligand 1 in sarcoma: a meta-analysis
Source: Oncotarget. 2017 Jul 11;8(35):59570–80. doi: 10.18632/oncotarget.19168 (PMC5601756; doi:10.18632/oncotarget.19168)
Supplement: Supplementary file 1 [file oncotarget-08-59570-s001.pdf]

Prognostic value of programmed death-ligand 1 in sarcoma: a meta-analysis

SUPPLEMENTARY MATERIALS

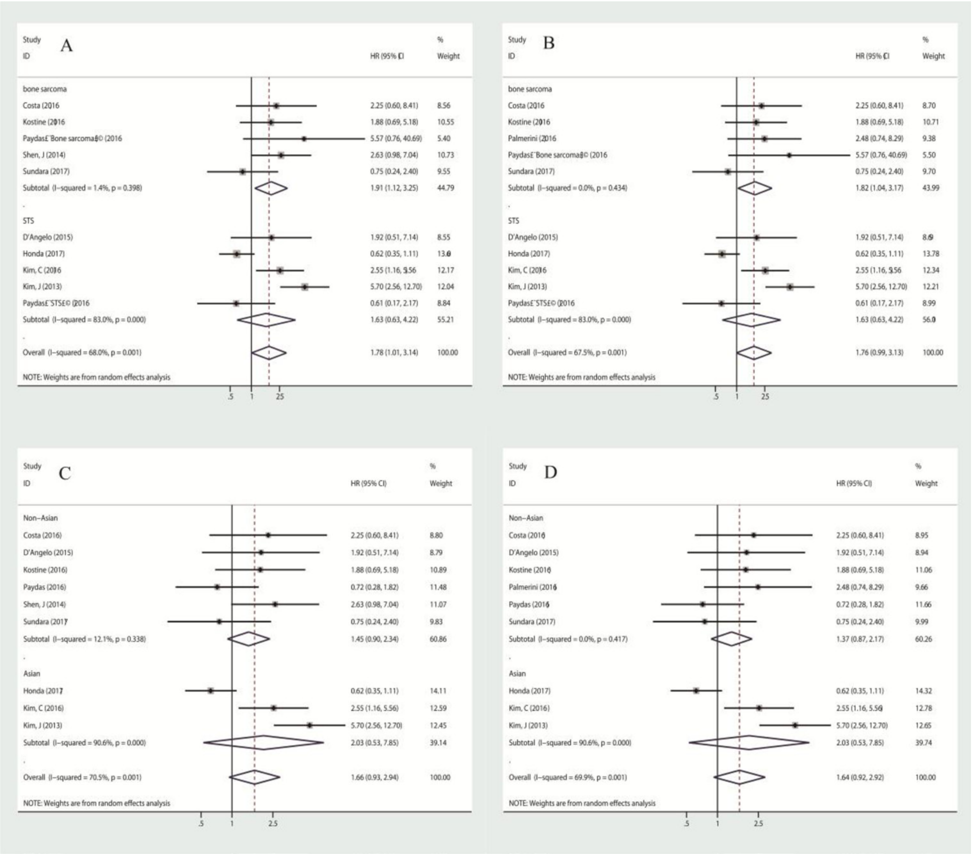

Supplementary Figure 1: Subgroup analysis of association between PD-L1 expression and overall survival stratified by histological subtype, when Palmerini's (**A**), or Shen J's (**B**) study was excluded; Subgroup analysis of association between PD-L1 expression and overall survival stratified by country, when Palmerini's (**C**) or Shen J's (**D**) study was excluded.

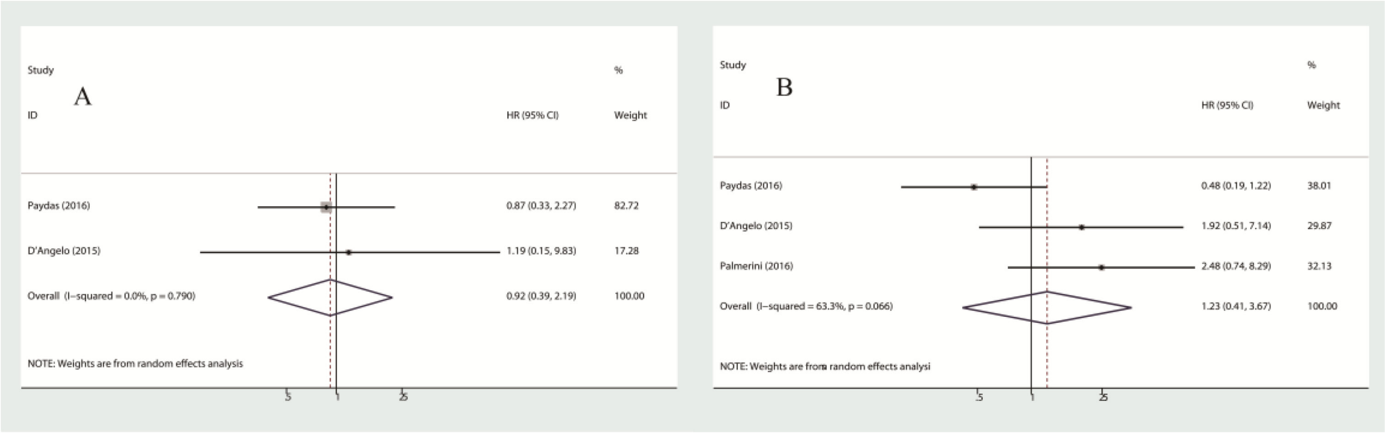

Supplementary Figure 2: Association between PD-L1 expression in tumor cells (**A**) or tumor microenvironment (**B**) and overall survival.

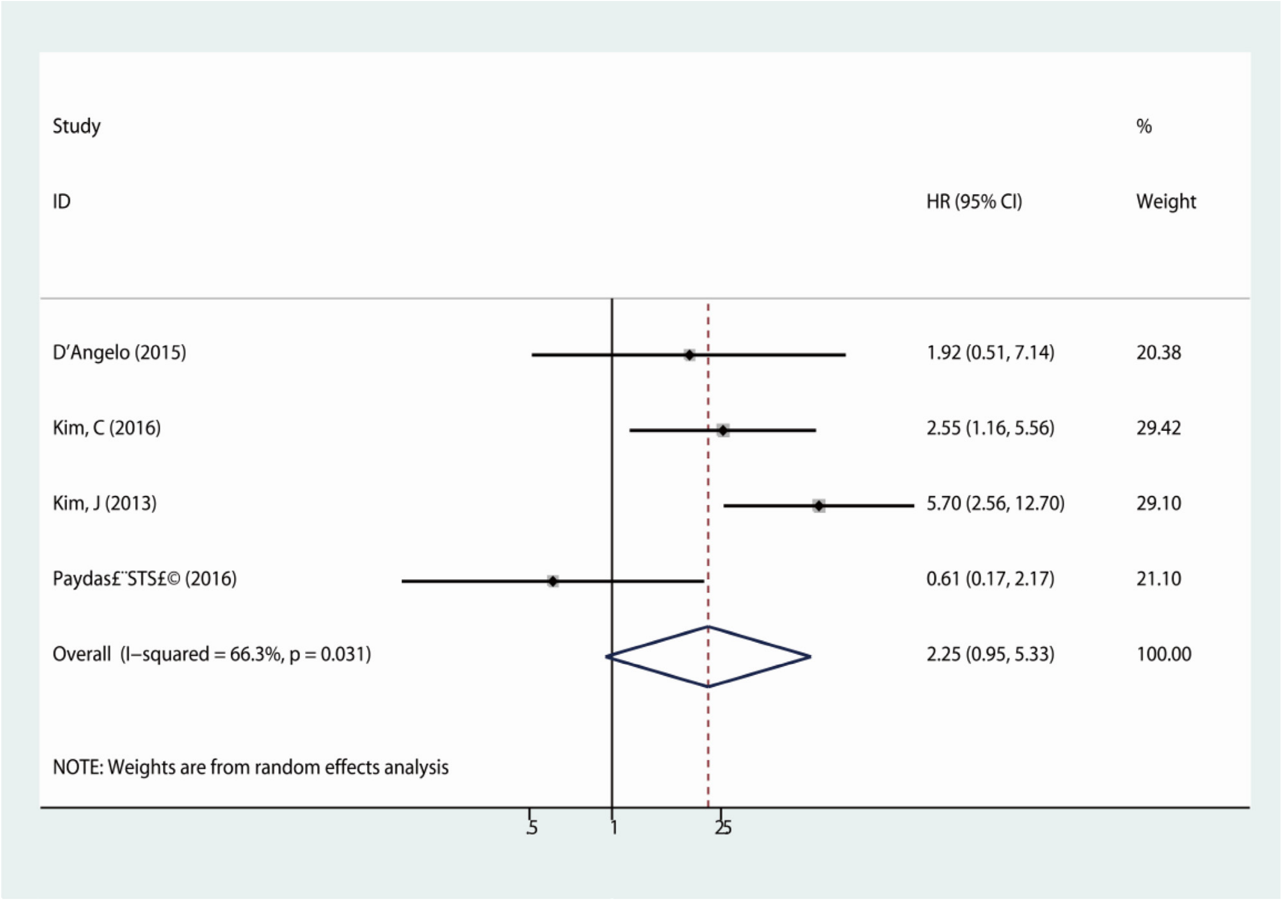

Supplementary Figure 3: Association between PD-L1 expression and overall survival for STS, when Honda's study was excluded.

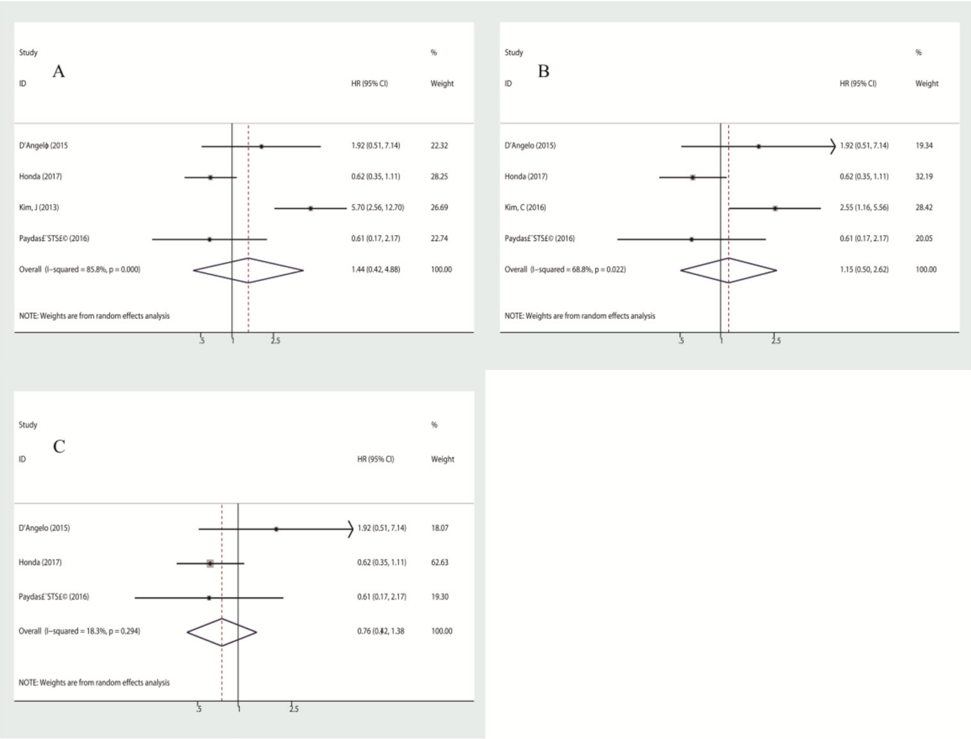

Supplementary Figure 4: Association between PD-L1 expression and overall survival for STS, when Kim C's study (**A**) or Kim J's study (**B**) or both of them (**C**) were excluded.
